# Dysfunctional GPR40/FFAR1 signaling exacerbates pain behavior in mice

**DOI:** 10.1371/journal.pone.0180610

**Published:** 2017-07-19

**Authors:** Kazuo Nakamoto, Fuka Aizawa, Kei Miyagi, Takuya Yamashita, Mitsumasa Mankura, Yutaka Koyama, Fumiyo Kasuya, Akira Hirasawa, Takashi Kurihara, Atsuro Miyata, Shogo Tokuyama

**Affiliations:** 1 Department of Clinical Pharmacy, School of Pharmaceutical Sciences, Kobe Gakuin University, Chuo-ku, Kobe, Japan; 2 Biochemical Toxicology Laboratory, Faculty of Pharmaceutical Sciences, Kobe Gakuin University, Chuo-ku, Kobe, Japan; 3 Faculty of Food Culture, Kurashiki Sakuyo University, Kurashiki, Okayama, Japan; 4 Laboratory of Pharmacology, Faculty of Pharmacy, Osaka Ohtani University, Tondabayashi, Osaka, Japan; 5 Department of Genomic Drug Discovery Science, Graduate School of Pharmaceutical Sciences, Kyoto University, Kyoto, Japan; 6 Department of Pharmacology, Graduate School of Medical and Dental Sciences, Kagoshima University, Kagoshima, Japan; University of Southern California, UNITED STATES

## Abstract

We previously showed that activation of G protein-coupled receptor 40/free fatty acid receptor 1 (GPR40/FFAR1) signaling modulates descending inhibition of pain. In this study, we investigated the involvement of fatty acid-GPR40/FFAR1 signaling in the transition from acute to chronic pain. We used GPR40/FFAR1-knockout (GPR40KO) mice and wild-type (WT) mice. A plantar incision was performed, and mechanical allodynia and thermal hyperalgesia were evaluated with a von Frey filament test and plantar test, respectively. Immunohistochemistry was used to localize GPR40/FFAR1, and the levels of free fatty acids in the hypothalamus were analyzed with liquid chromatography-tandem mass spectrometry. The repeated administration of GW1100, a GPR40/FFAR1 antagonist, exacerbated the incision-induced mechanical allodynia and significantly increased the levels of phosphorylated extracellular signal-regulated kinase in the spinal cord after low-threshold touch stimulation in the mice compared to vehicle-treated mice. The levels of long-chain free fatty acids, such as docosahexaenoic acid, oleic acid, and palmitate, which are GPR40/FFAR1 agonists, were significantly increased in the hypothalamus two days after the surgery compared to levels in the sham group. Furthermore, the incision-induced mechanical allodynia was exacerbated in the GPR40KO mice compared to the WT mice, while the response in the plantar test was not changed. These findings suggested that dysfunction of the GPR40/FFAR1 signaling pathway altered the endogenous pain control system and that this dysfunction might be associated with the development of chronic pain.

## Introduction

Even after a damaged or inflamed site that originally caused pain has been repaired, chronic pain can persist for months or years after surgery [1–4]. To date, the detailed mechanisms that underlie the development of chronic pain are not fully understood [5]. To improve the quality of life of patients with chronic pain, the development of innovative drugs satisfy patients and contribute to the treatment of chronic pain is necessary.

G protein-coupled receptor 40/free fatty acid (FFA) receptor 1 (GPR40/FFAR1) [6], which is activated by middle- to long-chain fatty acids, such as docosahexaenoic acid (DHA), is expressed abundantly in the central nervous system and pancreatic β-cells [7, 8]. Previously, we found that GPR40/FFAR1 is widely expressed in the brain [9, 10] and spinal cord [11] of rodents and that GPR40/FFAR1 agonists, such as DHA and GW9508, produce antinociceptive effects against chemical-, mechanical-, and thermal-induced pain stimuli. The results of other studies have suggested that brain GPR40/FFAR1 signaling is related to antidepressant effects [12] and the generation of newborn neurons in learning and memory [13–16].

We previously demonstrated that the intracerebroventricular (i.c.v.) administration of GW9508 or DHA reduces formalin-induced inflammatory pain by increasing β-endorphin release in the arcuate nucleus of hypothalamus and activating the opioidergic system [9] In addition, GW9508 or DHA suppresses complete Freund’s adjuvant-induced mechanical allodynia and thermal hyperalgesia, which indicates that these effects were due to the increased release of β-endorphin through the activation of pro-opiomelanocortin neurons [17]. These results suggest that hypothalamic GPR40/FFAR1 might be the key factor in modulating endogenous pain control systems. In addition, we have shown that GPR40/FFAR1 is found in the descending pain control neurons, such as serotonergic and noradrenergic neurons [18]. These neurons are directly or indirectly activated by injections of GPR40/FFAR1 agonists, which suggests that this signaling is involved in modulating the endogenous pain control system. However, the involvement of this signaling in the transition from acute to chronic pain is unclear. In this study, we used pharmacological techniques to examine the role of descending endogenous pain inhibitory system after incisional injury.

## Materials and methods

### Animals

The present study was performed in accordance with the Guiding Principles for the Care and Use of Laboratory Animals adopted by the Japanese Pharmacological Society. All of the experiments were approved by the Ethical Committee for Animal Experimentation of Kobe Gakuin University (approval number A16-23; Kobe, Japan). All animal studies were performed according to the ARRIVE guidelines as reported previously [19, 20]. A total of 144 mice were used in the experiments in this study.

**Fig 1:**
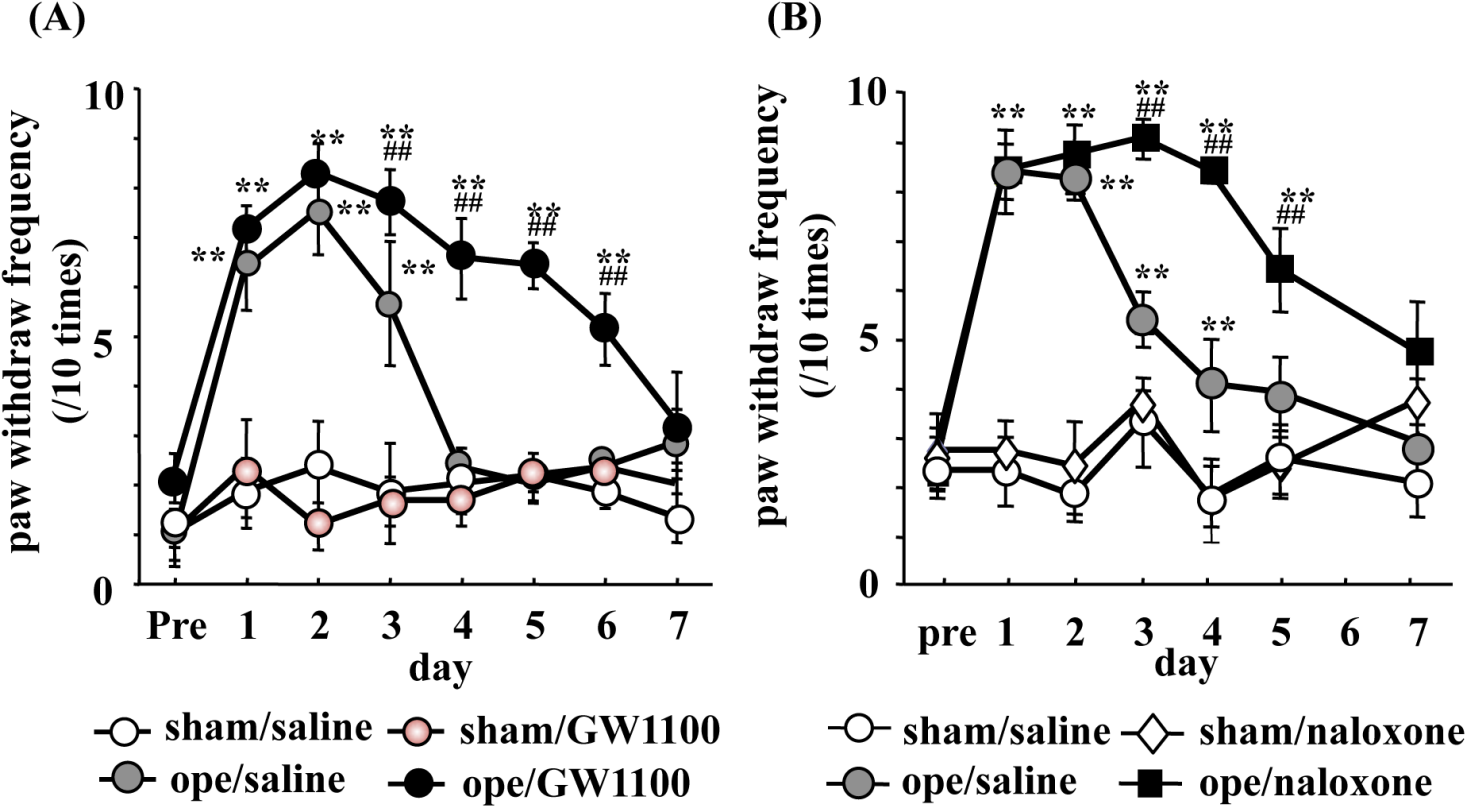
Effects of repeat administration of GW1100 or naloxone on incision-induced mechanical allodynia in the ipsilateral hind paw. The time course of mechanical allodynia in the postoperative pain model mice, with or without GW1100, a selective GPR40/FFAR1 antagonist (A), or naloxone, a non-selective opioid receptor antagonist (B). A plantar incision was performed in the mice as follows. A 1-cm longitudinal incision was made through the skin and fascia of the plantar foot. The underlying muscle was elevated with curved forceps, leaving the muscle origin and insertion intact. Ope indicates the postoperative pain model mice. The von Frey filament was applied to the middle of the plantar surface of the hind paw with a weight of 0.4 g. Data are presented as mean ± standard error of the mean (SEM). Sham/saline (n = 8), Sham/GW1100 (n = 8), Ope/saline (n = 8), Ope/GW1100 (n = 8). Sham/saline (n = 8), Sham/naloxone (n = 8), Ope/saline (n = 8), Ope/naloxone (n = 8). **significant difference vs. sham. ^##^significant difference vs. ope.

**Fig 2:**
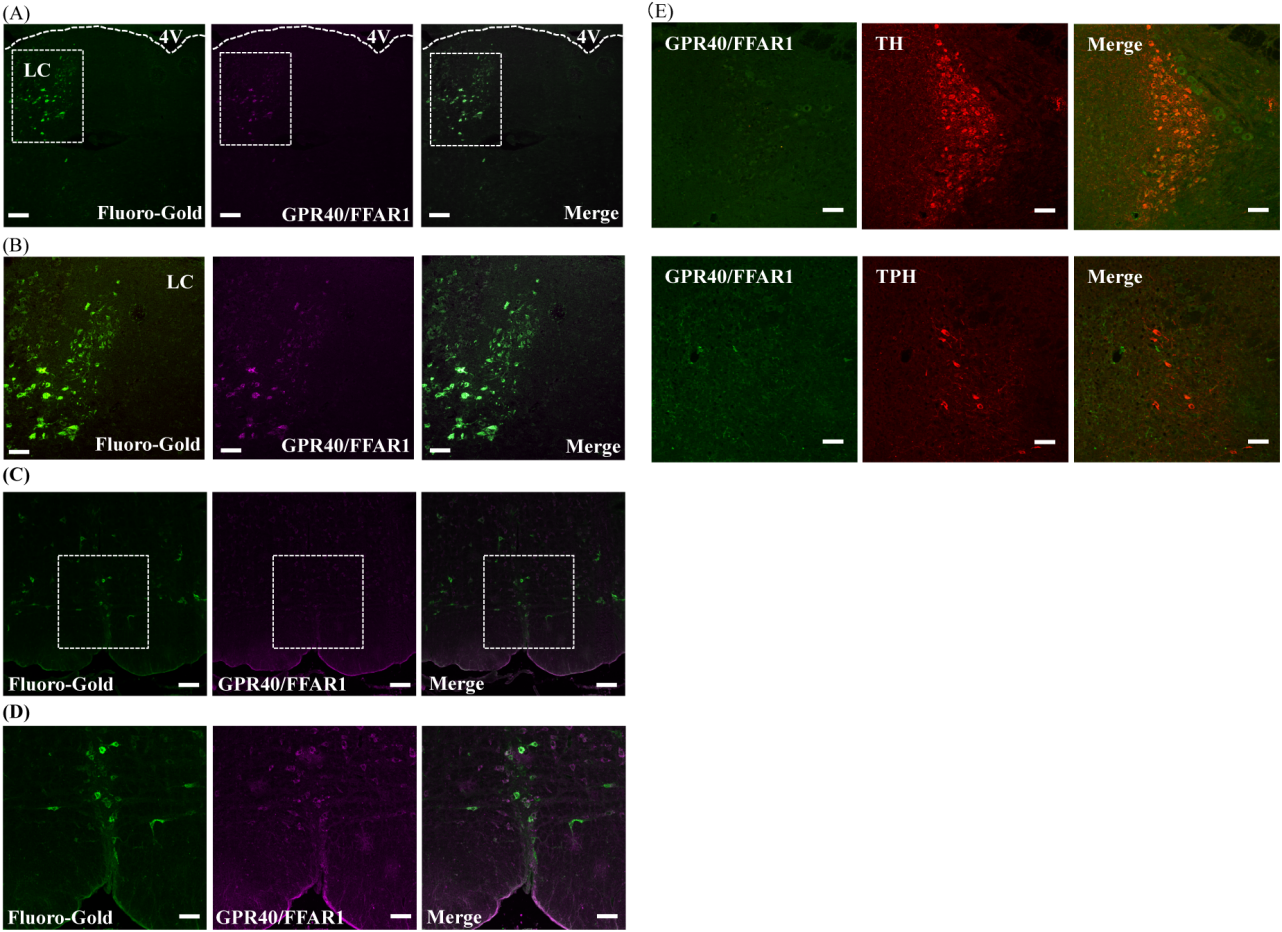
Localization of GPR40/FFAR1 and Fluoro-Gold in the locus coeruleus (LC) and rostral ventromedial medulla (RVM). Representative images of Fluoro-Gold (green) and GPR40/FFAR1 (magenta) staining in the LC (A, B) and RVM (C, D). 4% Fluoro-Gold was intrathecally (i.t.) administered into the L4—L5 region of the spinal cord of intact ddY mice. At 3 days after i.t. injection of 4% Fluoro-Gold, brain sections including the LC and RVM were prepared for double immunofluorescence study. Representative images are shown. Green; Fluoro-Gold positive cells, Magenta; GPR40/FFAR1 positive cells. Five mice were used in this study. Images (A, C); low-power field (Original magnification 10 x), Images (B, D); high-power field (Original magnification 20 x). Immunoreactivity for GPR40 in the LC and RVM of GPR40KO mice (E). Scale bars: 50 μm (Original magnification 20x), Abbreviations: 4V, fourth ventricle.

**Fig 3:**
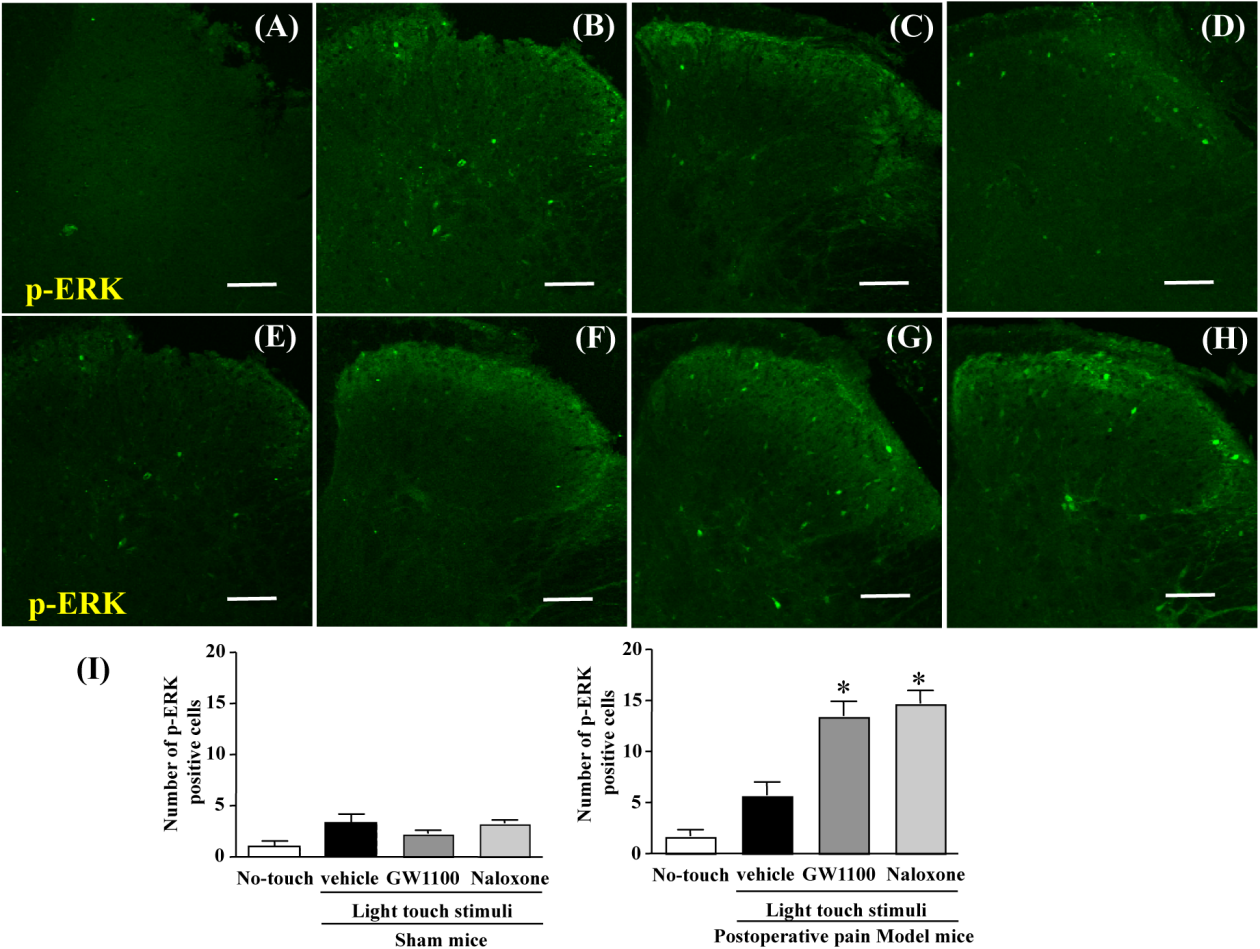
Effect of GW1100 or naloxone on spinal p-ERK expression induced by cotton bud stimulation. At 7 days after surgery, mice recovered from incision-induced pain. And then, GW1100 (10 μg), a selective GPR40/FFAR1 antagonist was intracerebroventricularly (i.c.v.) injected in mice at 15 min before light touch stimulation. Naloxone (1 mg/kg), a non-selective opioid receptor antagonist, was intraperitoneally administered in mice 30 min before light touch stimulation. A cotton tip was gently stroked across the plantar surface, once every 5 s for 5 min. After the end of this light touch stimulation, the mice’s spinal cords, including L4—L5 samples, were collected and, then, we performed an immunohistochemical study for p-ERK (green), which is a marker for neuronal activity. Representative images are shown. Scale bars: 50 μm (Original magnification 20x), A; Sham no-touch, B; Sham touch-vehicle, C; Sham touch-GW1100, D; Sham touch-Naloxone, E; Ope no-touch, F; Ope touch-vehicle, G Ope touch-GW1100, H; Ope touch-Naloxone. Twenty-four mice were used in this study. “Sham no touch” indicated mice without light touch stimulation. “Sham touch” mice with light touch stimulation. “Ope no touch” postoperative pain model mice without light touch stimulation. “Ope touch” postoperative pain model mice with light touch stimulation. (n = 3) *significant difference vs. Ope touch-vehicle.

**Fig 4:**
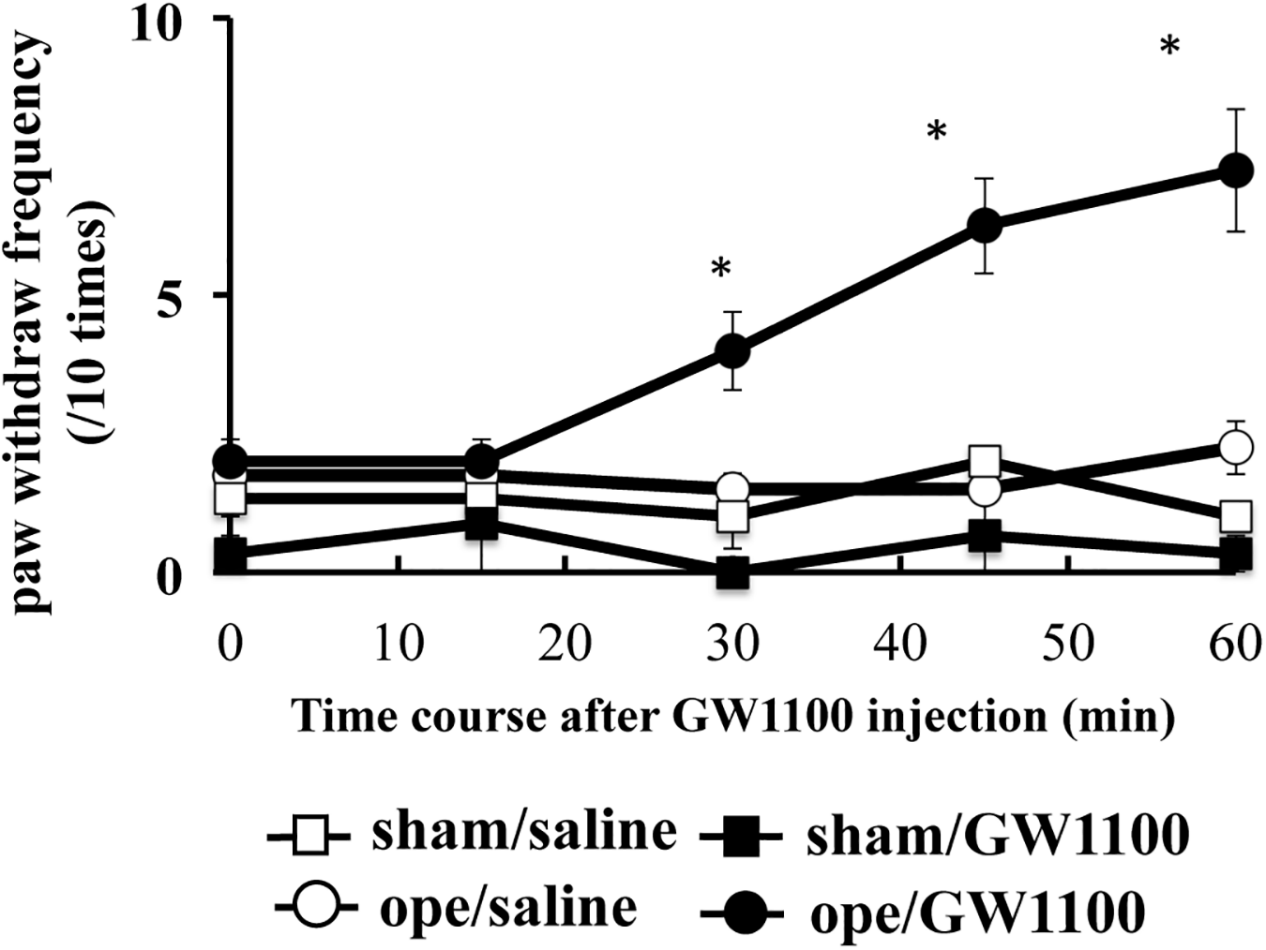
Effect of GW1100 on pain behavior mice recovered from incision-induced pain. At 7 days after surgery, mice were recovered from incision-induced pain. Then, GW1100, a selective GPR40/FFAR1 antagonist, or vehicle (4% DMSO) was i.c.v. injected in mice at 15 min prior to the von Frey test. The von Frey test was measured at 0, 15, 30, 45, and 60 min after i.c.v. injection. The von Frey filament was applied to the middle of the plantar surface of the hind paw with a weight of 0.4 g. Withdrawal responses, following hind paw stimulation, were measured 10 times and mechanical allodynia, defined as an increase in the number of withdrawal responses to the stimulation, was compared. *significant difference vs. vehicle. Data are presented as mean ± SEM. ddY mice were used in this experiment. Sham/saline (n = 6), Sham/GW1100 (n = 6), Ope/saline (n = 6), Ope/GW1100 (n = 6).

**Fig 5:**
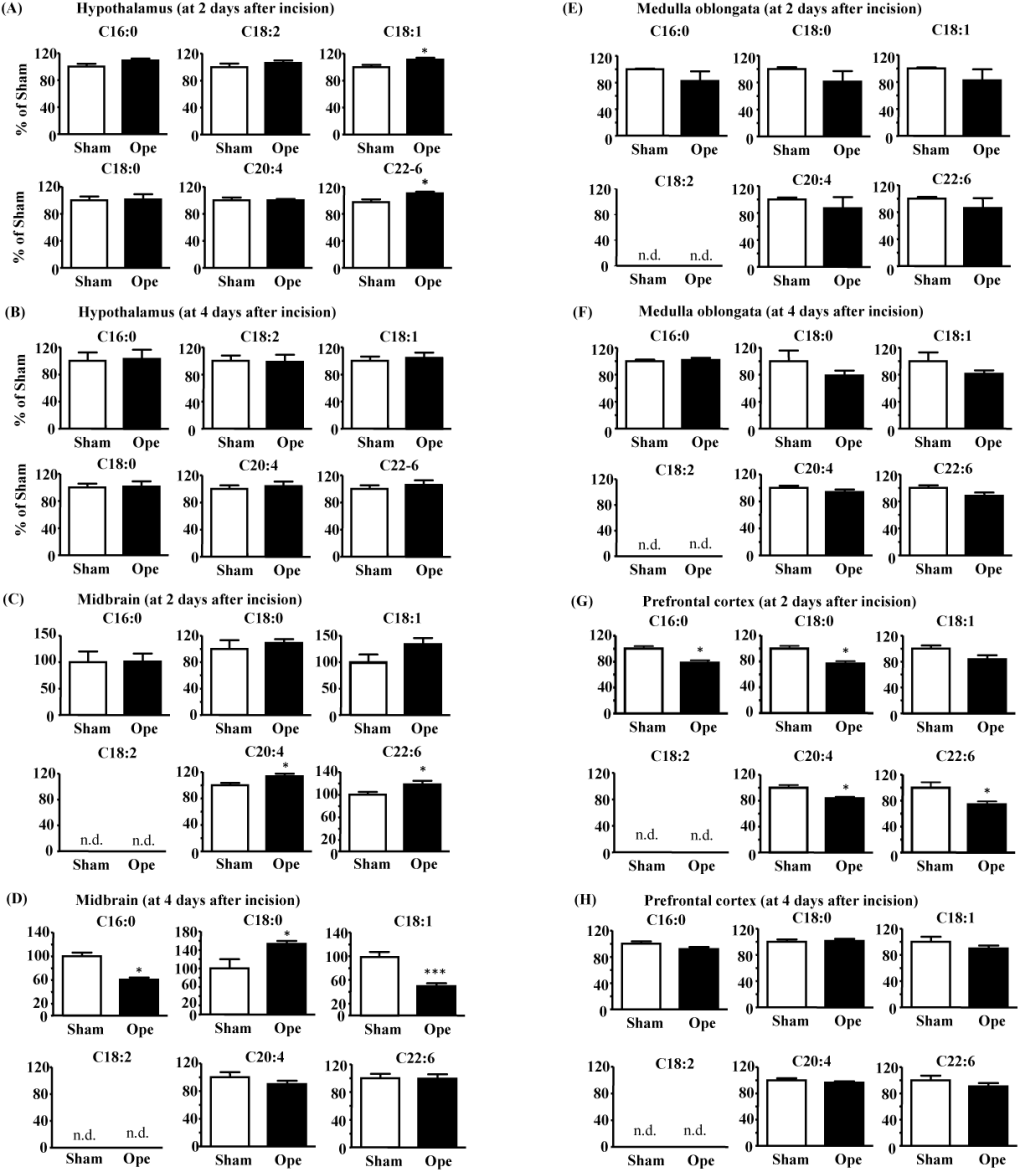
FFAs analysis in each brain tissue of postoperative pain model mice. FFAs were analyzed with UHPLC-MS/MS using multireaction monitoring; the FFA composition ratio of palmitic acid (C16:0), stearic acid (C18:0), oleic acid (C18:1), linolenic acid (C18:2), arachidonic acid (C20:4) and DHA (C22:6) in the hypothalamus (Fig 4A and 4B), midbrain (Fig 4C and 4D), medulla oblongata (Fig 4E and 4F) and prefrontal cortex tissues (Fig 4G and 4H) at days 2 (Fig 4A, 4C, 4E and 4G) and 4 (Fig 4B, 4D, 4F and 4H) after surgery. Data are presented as mean ± SEM. ddY mice were used in this experiment. Sham (2 days) (n = 8), Ope (2 days) (n = 8), Sham (4 days) (n = 8), Ope (4 days) (n = 8); * significant difference vs. sham (Student’s t-test).

**Fig 6:**
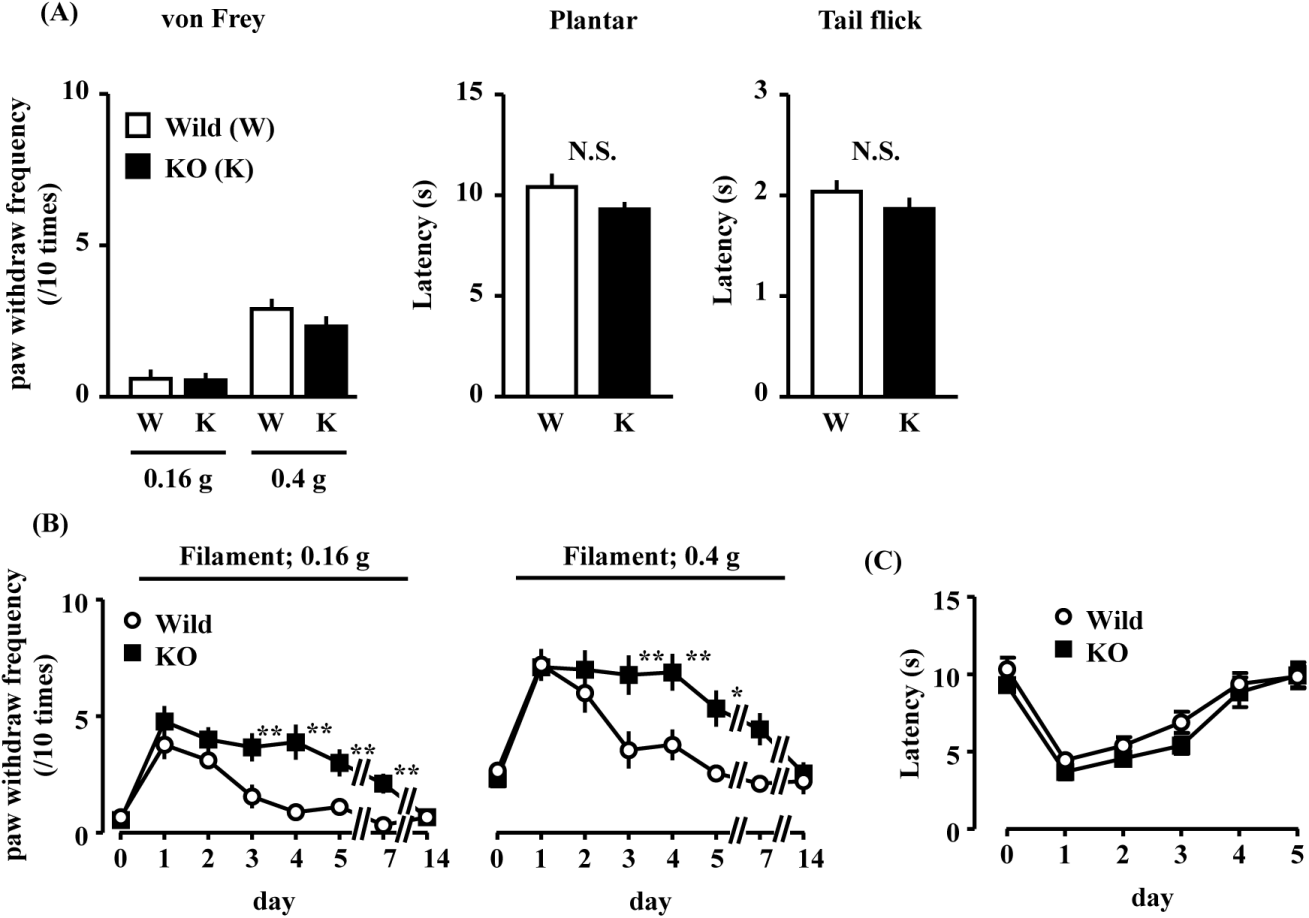
Effect of GPR40/FFAR1 knockout on basal mechanical allodynia and thermal hyperalgesia after incision. (A) Basal mechanical threshold in the von Frey, plantar, and tail-flick test. Time course of mechanical sensitivity (B) and thermal hyperalgesia (C) after incision. Each filament was applied to the right hind paw 10 times. Data are shown as mean ± SEM. C57BL/6J WT or GPR40KO mice were used in all experiments. (A) Wild type (Wild); n = 9, GPR40 knockout (KO); n = 9; *denotes significant difference identified with Student’s t-test. *, **denotes significant difference vs. paired wild type mouse identified with two-way repeated measures (time and genotype) analysis of variance, post-hoc test (Bonferroni). (A) The von Frey filament was applied to the middle of the plantar surface of the hind paw with a weight of 0.16 g or 0.4 g (A, B).

Male ddY (7 weeks old) and C57BL/6J (7 weeks old) mice were obtained from Japan SLC, Inc. (Hamamatsu, Japan). GPR40/FFAR1-knockout (GPR40KO) mice on a mixed C57BL/6/129 background were generated by homologous recombination in embryonic stem cells. Exon 1 of the *Ffar1* was replaced with a PGK-neo cassette. Frozen *Ffar1-*/*-* fertilized oocytes were inoculated into pseudopregnant foster mothers (ICR strain). The mice were backcrossed onto the C57BL/6 strain over nine generations. The pups were screened with polymerase chain reactions that were performed on the genomic DNA. Wild-type (WT) mice were used as the controls. Male ddY mice were used in the von Frey test, immunohistochemical study, and FFA analysis (Figs 1–5), whereas C57BL/6J and GPR40KO (C57BL/6J) mice were used in the plantar test, tail-flick test, and von Frey test (Fig 6). All of the behavior tests were performed by experimenters who were blind to the surgeries and genotype. The mice were housed in cages at 23–24°C with a 12-h light-dark cycle (lights on from 8 am to 8 pm), with food and water available *ad libitum*.

### Drug administration

A selective GPR40/FFAR1 antagonist, GW1100 (50 mg; Cayman Chemical, Ann Arbor, MI, USA), was dissolved in 100% dimethyl sulfoxide (DMSO; Sigma-Aldrich Japan K.K., Tokyo, Japan), and the solution was diluted with saline (4% DMSO, final concentration). The concentrations of GW1100 were selected based upon our previous publications [18]. GW1100 (10 μg) was administered through the i.c.v. route once a day for 5 days. At thirty min after the i.c.v. injection of GW1100, the von Frey test was performed in the mice. Mice that do not receive GW1100 were i.c.v. administrated to 4% DMSO as a control. Naloxone (1 mg/kg), a non-selective opioid receptor antagonist, was intraperitoneally administered in mice once a day for 5 days or at 30 min before light touch stimulation. Experimenters were blinded to drug administration in all behavioral tests. As described in detail previously [18], the mice were anesthetized with sodium pentobarbital (65 mg kg^-1^) so a hole could be made in the skull for the injections, and a needle (26-gauge, Natsume Seisakusyo Co., Ltd., Tokyo, Japan) that was attached to a 50 μL Hamilton microsyringe was inserted into a unilateral injection site. The drugs were administered in a volume of 5 μL through a disposable 27-gauge needle [21] under non-anesthesia. The needle was inserted perpendicular to the skull. Each solution was injected without the use of a cannula. While anesthetized, some groups of mice were administered an intrathecal (i.t.) injection of 4% Fluoro-Gold (Abcam plc, Tokyo, Japan) or saline. For the i.t. injections, 4% Fluoro-Gold were administrated in a volume of 5 μL through a disposable 27-gauge needle, which was inserted into the subarachnoid space through the intervertebral foramen between L4 and L5 [22]. Seventy-two hours after the 4% Fluoro-Gold injection, brain sections that included the rostral ventromedial medulla (RVM) and locus coeruleus (LC) were collected, and we performed a double immunofluorescence study to determine the colocalization of Fluoro-Gold and GPR40/FFAR1.

### Postoperative pain mouse model

A plantar incision was performed in the mice, as described previously [23]. In brief, the mice were anesthetized with sodium pentobarbital (65 mg kg^-1^). After antiseptic preparation of the left hindpaw, a 1-cm longitudinal incision was made through the skin and fascia of the plantar foot. The underlying muscle was elevated with curved forceps, leaving the muscle origin and insertion intact. The sham mice were anesthetized with sodium pentobarbital (65 mg kg^-1^) only.

### von Frey test

As described in detail previously [17], mechanical allodynia was assessed with von Frey filaments (NeuroScience Inc., Osceola, WI, USA). The mice were placed on a 5 × 5-mm wire-mesh grid floor that was covered with a foil-wrapped cup to avoid visual stimulation and allowed to adapt for 3 h prior to the von Frey test. Then, the von Frey filament was applied to the middle of the plantar surface of the hind paw with 0.4 g (Figs 1A, 1B, 4 and 6B) or 0.16 g (Fig 6A). The withdrawal responses following the hind paw stimulation were measured 10 times, and the mechanical allodynia, which was defined as an increase in the number of withdrawal responses to the stimulation, was compared [24].

### Light touch stimulation

A light touch stimulus was applied manually with a cotton tip to the ventral surface of the hindpaw once every 5 s for 5 min according to the methods previously described [25].

### Plantar test

The thermal hyperalgesia of the hind paw was estimated with the plantar test (Ugo Basile Srl, Varese, Italy) as reported previously [17]. Briefly, the mice were acclimatized to an apparatus consisting of individual Perspex boxes on an elevated glass table, and an infrared radiant heat (40 W) source was directed onto the plantar surface of the hind paw, with the withdrawal response defined as the paw withdrawal latency. The heat application cut-off point was set at 20 s to prevent tissue damage. In order to give a paw withdrawal latency of ∼10 s in the intact mice, the apparatus was calibrated.

### Tail-flick test

As described in detail previously [26], the antinociceptive responses against thermal stimuli were evaluated with the tail-flick test. The mice were gently held with their tail positioned in the tail-flick apparatus (MK-330B; Muromachi Kikai Co., Ltd., Tokyo, Japan) for the radiant thermal stimulation of the dorsal surface of the tail. The intensity of the heat stimuli was set to cause the animal to flick its tail within 3 s as the baseline of the tail-flick latency. The cut-off time was set at 10 s to minimize tissue damage.

### Brain and spinal cord tissue preparation

As described in detail previously [18], the mice were deeply anesthetized with sodium pentobarbital (65 mg kg^-1^) and perfused transcardially with 0.1 M phosphate-buffered saline (PBS, pH 7.4), which was followed by 4% paraformaldehyde in 0.1 M PBS (pH 7.4). The brain and spinal cord (L4-L5) sections were collected, postfixed in 4% paraformaldehyde for 3 h, cryoprotected in 10% sucrose at 4°C for 3 h, and then placed in 20% sucrose at 4°C overnight. The following day, the tissues were frozen in optimal cutting temperature compound (Tissue-Tek OCT Compound, Sakura Finetek Japan, Co., Ltd., Tokyo, Japan) and stored at -80°C until use. Sample sections (15-μm thick) were cut with a cryostat (CM1850, Leica Microsystems GmbH, Wetzlar, Germany) and mounted on a MAS-coated glass slide (S9115, Matsunami Glass Ind., Ltd., Osaka, Japan).

### Immunofluorescence labeling

The double immunofluorescence studies were performed according to methods described previously [18]. The brain and spinal cord sections were incubated with blocking buffer that and incubated for 1 hour in PBS containing 10% (v/v) normal goat serum containing 1% (w/v) bovine serum albumin (BSA) at room temperature. The brain and spinal cord sections were then incubated with a rabbit polyclonal anti-GPR40 antibody (1:10000; Trans Genic Inc., Fukuoka, Japan), rabbit polyclonal anti-Fluoro-Gold antibody (1:200, Merck Millipore Corporation, Tokyo, Japan), chicken polyclonal anti-tyrosine hydroxylase (TH) (1:200; Abcam plc), tryptophan hydroxylase, sheep polyclonal anti-TPH (TPH) (1:300; EMD Millipore Corporation) or rabbit polyclonal anti-phosphorylated extracellular signal-regulated kinase (p-ERK; 1:1,000, Cell Signaling Technology, Inc., Danvers, MA, USA), which were diluted in reaction buffer (PBS containing 10% (v/v) normal goat serum containing 1% (w/v) BSA), for 48 h at 4°C. The sections were incubated in secondary antibodies that were conjugated with AlexaFluor 488 and/or 594 (donkey polyclonal anti-rabbit IgG, donkey polyclonal anti-sheep IgG, donkey polyclonal anti-chicken IgG; or goat polyclonal anti-mouse IgG; 1:200; Thermo Fisher Scientific Inc., Waltham, MA, USA) at room temperature for 2 h. Immunoreactivity was detected with a confocal fluorescence microscope (FV1000, Olympus Corporation, Tokyo, Japan). For the spinal cord, the number of p-ERK-positive cells in three slices per animal were determined from a 500 × 500 μm^2^ area, and then the average count (three sections) for each treated subject was calculated. No staining was detected in the control sections when the corresponding primary or secondary antibody was omitted.

### Comparative FFA analyses

As described in detail previously [17, 27], the wet weights of the hypothalamic, midbrain, medulla oblongata and prefrontal cortex tissues were measured, and the tissue was homogenized in an internal standard solution. The samples were centrifuged at 15,000 × *g* for 15 min. The supernatant was collected and filtrated in a sample vial for the liquid chromatography-tandem mass spectrometry analysis. For the quantitation, we used a QTRAP 4500 (AB Sciex LLC, Framingham, MA, USA). We then operated the mass spectrometer in the negative-ion mode. The FFAs were quantified with selective multireaction monitoring in the negative ionization mode. The specific parameters were the following: ion spray voltage, -4,500 V; source temperature, 300°C; declustering potential range, -70 to -105 V; and collision energy range, -10 to -22 eV for the fragment ions. The peak of each FFA was monitored with the product ion that was obtained from the [M-H]- ion (i.e., m/z 255 → m/z 255 for palmitic acid, m/z 279 → m/z 279 for alpha-linoleic acid, m/z 281 → m/z 281 for oleic acid, m/z 283 → m/z 283 for stearic acid, m/z 303 → m/z 303 for arachidonic acid, and m/z 327 → m/z 327 for DHA). The concentration of each FFA was assessed in each calibration curve with the absolute calibration curve method.

### Statistical analyses

All of the data were analyzed with GraphPad Prism, version 4.0 (GraphPad Software, Inc., La Jolla, CA, USA). For the comparisons of two groups (Sham vs. Ope or GPR40KO mice vs. WT mice), Student’s unpaired *t*-tests were applied (behavioral test and FFA analysis). For multiple comparisons, one-way or two-way analysis of variance was used, which was followed by Bonferroni’s post hoc test to determine the individual group differences. The data are presented as the mean ± standard error of the mean. P values less than 0.05 were considered significant.

## Results

### Repeat administration of GW1100 or naloxone exacerbated incision-induced mechanical allodynia

One day after the surgery, the plantar incision-treated mice exhibited significantly increased responses to the mechanical stimuli of the ipsilateral hind paw. The incision-induced pain lasted four days, and the paw withdrawal threshold returned to baseline levels five days after the surgery. The paw withdrawal threshold in the ipsilateral hind paw of the incision-plus-GW1100-treated mice exhibited increased responses to the mechanical stimuli and did not return to baseline levels until seven days after the surgery (Fig 1A, Operation (Ope) × time interaction: F (18,84) = 3.44, p < 0.0001), Ope effect: F (6,84) = 6.709), p < 0.0001, time effect: F (3, 84) = 3.784, p < 0.0001). Neither sham surgery nor sham-plus-GW1100 treatment affected the paw withdrawal threshold in the ipsilateral hind paw. In contrast, the paw withdrawal threshold in the ipsilateral hind paw of the incision-plus-naloxone-treated mice did not return to baseline levels until seven days after the surgery, and the incision-plus-naloxone-treated mice had increased responses to the mechanical stimuli compared to the incision-only group (Fig 1B, Ope×time interaction: F (21,168) = 4.434, p < 0.0001), Ope effect: F (7,168) = 13.59, p < 0.0001, time effect: F (3, 168) = 77.17, p < 0.0001).

### Fluoro-Gold-positive cells in the LC and RVM were colocalized with GPR40/FFAR1

GPR40/FFAR1 was expressed in the LC of the pons and RVM of the medulla oblongata in WT mice (Fig 2A and 2B). Similar to GPR40/FFAR1, immunoreactivity for Fluoro-Gold, which is a retrograde tracer, was also observed in the LC of the pons and RVM of the medulla oblongata. GPR40/FFAR1 staining was colocalized with Fluoro-Gold staining in the LC of the pons (Fig 2A and 2B) and RVM (Fig 2C and 2D) of the medulla oblongata. In GPR40KO mice, immunoreactivity for GPR40 was not observed (Fig 2E).

### GW1100 or naloxone increased spinal p-ERK expression after light touch stimulation

In sham mice, positive cells of p-ERK in the dorsal horn of the spinal cord were barely observed after light touch. However, spinal p-ERK expression did not change between groups (Fig 3A–3D and 3I). In contrast, in mice that recovered from postoperative pain, the GW1100-treated or naloxone-treated mice exhibited significantly increased p-ERK protein expression after light touch stimulation in the surface layer of the spinal cord compared to those in the vehicle-treated or no-touch group (Fig 3E–3I).

### Mice that recovered from the incision-induced pain exhibited reinduced pain behavior in response to i.c.v. injection of GW1100

Mice showed gradually increased response time to mechanical stimulation after i.c.v. injection of GW1100, as opposed to vehicle-treated mice. These increases continued up to 60 min after GW1100 injection (Fig 4, Ope × time interaction: F (4,30) = 7.025, p < 0.001), Ope effect: F (4,30) = 8.418), p < 0.001, time effect: F (1, 30) = 42.64, p < 0.0001). In contrast, in uninjured sham mice, GW1100 did not affect the response against mechanical stimuli.

### The levels of several FFAs increased in each brain tissue of the postoperative pain model mice

At two days after the incision, the levels of oleic acid and DHA were significantly increased in the hypothalamic area compared to sham mice (Fig 5A, oleic acid, Sham, 100 ± 3.5, Ope 2 days, 111. 2 ± 3.0; DHA, Sham, 100 ± 4.4, Ope 2 days, 110.4 ± 2.5). Similarly, in the midbrain area, oleic acid, arachidonic acid, and DHA levels were also significantly increased (Fig 5B, oleic acid, Sham, 100 ± 14.9, Ope 2 days, 134. 2 ± 11.9; arachidonic acid, Sham, 100 ± 3.8, Ope 2 days, 113.8 ± 4.0; DHA, Sham, 100 ± 5.1, Ope 2 days, 118.3 ± 6.8). However, FFAs in the region of the medulla oblongata did not change between Sham and Ope mice (Fig 5C). Furthermore, in the prefrontal cortex area, all FFAs levels were significantly decreased compared to sham mice (Fig 5D, palmitic acid, Sham, 100 ± 4.2, Ope 2 days, 78.0 ± 4.2; oleic acid, Sham, 100 ± 5.0, Ope 2 days, 83.6 ± 6.4; stearic acid, Sham, 100 ± 4.3, Ope 2 days, 76.6 ± 4.0; arachidonic acid, Sham, 100 ± 4.3, Ope 2 days, 83.7 ± 2.5; DHA, Sham, 100 ± 8.7, Ope 2 days, 74.4 ± 4.8).

In contrast, at four days after the incision, the levels of all FFAs in the hypothalamus and prefrontal cortex did not change between Sham and Ope (Fig 5A and 5D). In the midbrain, palmitic and oleic acid were significantly decreased (Fig 5B, palmitic acid, Sham, 100 ± 6.5, Ope 4 days, 60.6 ± 3.7; oleic acid, Sham, 100 ± 7.6, Ope 4 days, 49.4 ± 5.1) compared to sham mice, while stearic acid was significantly increased (stearic acid, Sham, 100 ± 20.3, Ope 4 days, 153.5 ± 6.6). Moreover, stearic and oleic acid were significantly decreased in the medulla oblongata compared to sham mice (Fig 5C). Changes in the other FFAs were not observed in each brain region after the incision. Linolenic acid was not detected in the midbrain, medulla oblongata, and prefrontal cortex at 2 and 4 days after paw incision (Fig 5A–5D).

### GPR40/FFAR1-deficient mice exhibited exacerbated mechanical allodynia after paw incision, while thermal hyperalgesia was unaffected

Intact WT and GPR40KO mice did not show susceptibility to low-threshold stimuli, such as von Frey filaments (0.16 g and 0.4 g, Fig 6A), or to thermal pain stimulation, including the plantar and tail-flick tests. WT mice had significantly increased responses to low-threshold stimuli one day after the paw incision. These increases peaked two days after the paw incision, and they recovered to basal levels four days after the paw incision. In contrast, GPR40KO mice exhibited significantly increased responses to mechanical stimuli compared to those of the sham surgery mice, and the responses did not return to baseline levels until 14 days after the surgery (Fig 6B).

## Discussion

In this study, we examined whether the inhibition of endogenous pain control, through the GPR40/FFAR1 signaling system, prolonged incision-induced pain behavior and promoted the transition from acute to chronic pain after the surgery. We found that repeat administration of the GPR40/FFAR1 antagonist GW1100 or naloxone exacerbated mechanical allodynia that was caused by postoperative pain, which suggested that the mechanism underlying pain exacerbation induced by inhibition of GPR40/FFAR1 may be associated with naloxone-induced exacerbation of postoperative pain. These results are in agreement with our previous findings that i.c.v. pretreatment with GW1100 significantly exacerbates pain-like behavior in the late phase of the formalin test [18].

Currently, the neuropathological mechanisms underlying the transition from acute to chronic pain are complicated and poorly understood [4]. A recent study has shown that μ-opioid receptor agonist-induced antinociceptive effects are not the result of continuous opioid release but rather attributable to the constitutive activity of μ-opioid receptors [28, 29]. These physiological phenomena were also shown in a recent human study [30], and they are known as latent sensitization, which exhibits key features of chronic pain [31]. Latent sensitization may be induced by a wide variety of painful stimuli, including paw incisions [32, 33] and inflammation [28], and these noxious stimuli may lead to a period of hyperalgesia that ranges from several days to months [34]. Furthermore, we demonstrated that repeat administration of naloxone delayed the recovery from incision-induced mechanical allodynia and that GW1100 exacerbated the pain behavior in mice that had recovered from incision-induced mechanical allodynia. Our results and those of previous reports indicate that hyperalgesia may still be present, even after the pain has recovered, but it might be suppressed by compensatory activation of the opioid receptors. Therefore, we have revealed one possible mechanism: that activation of GPR40/FFAR1 downstream pathways during pain may activate an endogenous pain control system and contribute to the stage of pain recovery, but not to early stages of pain.

Descending noradrenergic neurons that originate from the LC and serotonergic neurons that originate from the RVM suppress nociceptive transmission [35, 36]. Damage of the descending inhibition and augmentation of the facilitation during chronic pain increase nociceptive transduction at the spinal cord [37]. Therefore, the modulation of descending pain control systems is thought to play an important role in the pathology of pain. In this study, we performed tracing experiments, with the retrograde tracer Fluoro-Gold, to determine whether GPR40/FFAR1 was colocalized with Fluoro-Gold-positive cells. Fluoro-Gold labeling was observed on descending-pain-control-related neurons, including serotonergic neurons in the RVM of the medulla oblongata and noradrenergic neurons in the LC of the pons. The results of the double-immunofluorescence study support the findings of our previous reports that GPR40/FFAR1 is colocalized on serotonergic and noradrenergic neurons [18]. These results suggest that GPR40/FFAR1 in the RVM and LC could mediate activation of the descending endogenous pain inhibitory system after incisional injury.

p-ERK is a marker for neuronal activation, which induces the firing of action potentials by external stimuli such as pain or inflammation. [25]. For example, in the normal state, noxious chemical stimuli and the activation of C- and Aδ fibers by electrical stimuli activate ERK. In contrast, innocuous stimuli, such as the electrical stimulation of Aβ-fibers, light touch with a cotton puff, and warm water, do not activate ERK [38]. However, Gao et al. showed that light touch stimulation increases p-ERK expression in dorsal horn neurons in the inflammatory paw or after nerve injury [25]. After light touch stimulation, the GW1100-treated mice exhibited an increased number of p-ERK-positive cells in the spinal cord compared to the incision-only mice, which indicated that increase of spinal p-ERK expression, which is induced by inhibition of GPR40/FFAR1, may be relevant to pain exacerbation against mechanical allodynia.

In our previous study, increased levels of FFAs were observed in the hypothalamus of a complete Freund’s adjuvant-induced inflammatory pain model mice [17], which suggested that FFAs are continuously released during painful stimuli. To confirm whether FFAs in each brain area are affected by pain stimuli, we measured FFAs in the prefrontal cortex, midbrain, medulla oblongata, and hypothalamus of the brain, with or without postoperative pain, by using LC-MS/MS. We found that several FFAs including DHA increased in the midbrain and hypothalamus area, but not the medulla oblongata and prefrontal cortex, 2 days after incision. It is well known that the midbrain area, including the periaqueductal gray (PAG) or LC of the pons, is related to the modulation of pain [18]. Moreover, the hypothalamic area is related to the production of β-endorphin, which is one of the opioid peptides [9]. These changes of FFAs levels returned to baseline 4 days after paw incision, when mechanical hypersensitivity was recovered. Furthermore, an increase in FFAs levels, which was observed in this study was of a few percent points. However, it is proposed that these changes are important due to the deletion or inhibition of the FFAs-GPR40 signal causing exacerbation of pain after paw incision in this study. This is the first report concerning FFAs change in several brain regions during pain. Therefore, our results indicate that FFAs may be continuously released to suppress these phenomena when pain or inflammation signals are transduced to the brain. Our findings indicate that the levels of FFAs in each local brain area were increased in the early phase of postoperative pain, and these changes may contribute to pain modulation. However, it remains unclear why the pain behaviors exhibited two days after the paw incisions did not differ between the GW1100-treated and vehicle-treated mice, even though the levels of several FFAs were increased at this stage.

Interestingly, the GPR40KO mice showed exacerbated mechanical allodynia, but not thermal hyperalgesia. In contrast, in the absence of pain, this gene knockout had no effect on several pain tests, such as the von Frey, plantar, and tail-flick test. It is suggested that the changes in this signaling system are more likely to have been induced after painful stimulation, such as paw incision. In fact, the results of our previous study showed that the GPR40/FFAR1 agonist GW9508 increased spinal noradrenaline and serotonin levels in the presence of pain that was induced by formalin but not in the innocuous state [18]. These results indicate that the effect of GPR40/FFAR1 signaling may be limited to the regulation of tactile stimuli after paw incision. However, at this stage, we cannot explain why GPR40KO mice showed a different type of pain after paw incision. As a next step, further study is required to clarify the phenotypic differences between WT and GPR40KO mice. Furthermore, it is reported that mice submitted to receptor gene deletion might develop compensator mechanisms. For example, absence of gene at all stages of ontogenesis of mice may interfere with the normal developmental program and/or the organism may undergo changes in other system to compensate for gene absence [39]. Therefore, we cannot exclude the possibility of changes in other system to compensate for gene absence. However, mechanical sensitivity of uninjured GPR40KO mice similar to that observed in uninjured WT mice. Therefore, we suggest that there is no different between WT and GPR40KO mice in at least mechanical pain sensitivity.

However, it remains unclear why the GPR40KO mice did not affect the incision-induced thermal hyperalgesia, although they showed a tendency to decrease the withdrawal threshold against thermal stimuli. These results suggest that the supraspinal GPR40/FFAR1 signaling system may be more strongly activated by the release of FFAs during painful stimuli, and that this signaling activation might facilitate the descending inhibition system. Therefore, our findings suggest that the deletion of the GPR40/FFAR1 gene and, consequently, its signaling may induce dysfunction of the endogenous pain control system and result in an exacerbation of pain behavior.

In summary, we found that, in the early phase of pain, the levels of FFAs, including DHA, arachidonic acid, and oleic acid, significantly increased in the hypothalamus and midbrain area, which is related to the transduction pathway of pain. Furthermore, the inhibition of GPR40/FFAR1 signaling in knockout mice exacerbated mechanical allodynia due to incision-induced postoperative pain. Finally, our findings suggested that, in the pain state, GPR40/FFAR1 signaling may play a key role in the modulation of the endogenous pain control system and that GPR40/FFAR1 signaling could be an important factor facilitating recovery of pain.
